# Anti‐inflammatory effect of mesenchymal stem cells on hepatocellular carcinoma in the xenograft mice model

**DOI:** 10.1002/vms3.886

**Published:** 2022-07-15

**Authors:** Saieh Hajighasemlou, Mohsen Nikbakht, Saeedreza Pakzad, Abdolnaser Azadbakht, Samad Muhammadnejad, Milad Mirmoghtadaei, Safoora Gharibzadeh, Iman Seyhoun, Javad Verdi

**Affiliations:** ^1^ Department of Tissue Engineering & Applied Cell Sciences Tehran University of Medical Sciences (TUMS) Tehran Iran; ^2^ Food and Drug Administration Ministry of Health and Medical Education Tehran Iran; ^3^ Hematology, Oncology & Stem Cell Transplantation Research Center Tehran University of Medical Sciences (TUMS) Tehran Iran; ^4^ Department of Biomedical Engineering, Central Tehran Branch Islamic Azad university Tehran Iran; ^5^ Stem cells Research Center, Tissue Engineering and Regenerative Medicine Institute, Central Tehran Branch Islamic Azad university Tehran Iran; ^6^ Gene Therapy Research Center, Digestive Diseases Research Institute Tehran University of Medical Sciences Tehran Iran; ^7^ Immunology, Asthma and Allergy Research Institute Tehran University of Medical Sciences (TUMS) Tehran Iran; ^8^ Children's Medical Center, Pediatrics Center of Excellence Tehran University of Medical Sciences (TUMS) Tehran Iran; ^9^ Department of Epidemiology and Biostatistics Research Centre for Emerging and Reemerging Infectious Diseases Pasteur institute Tehran Iran

**Keywords:** hepatocellular carcinoma, human placenta, inflammation, mesenchymal stem cells

## Abstract

**Background:**

Hepatocellular carcinoma (HCC) is the fifth most diagnosed cancer and the second leading cause of cancer‐related deaths worldwide. Sorafenib is the standard treatment used in the advanced stages of HCC. Cell therapy with mesenchymal stem cells (MSCs)‐based cell therapy has proven effective in immune regulation and tumour growth inhibition.

**Objectives:**

In this study, we investigated the anti‐inflammatory effect of MSCs on HCC xenografts.

**Methods:**

Human HepG2 cell lines were subcutaneously implanted into the flank of 12 nude mice, divided into three groups: the control group, the IV group (intravenous MSCs injection) and the local group (local MSCs injection). Mice were sacrificed 6 weeks after tumour implantation, and tumours were resected entirety. Quantitative real‐time polymerase chain reaction (qRT‐PCR) measured the gene expression of inflammatory markers, including tumour necrosis factor‐α (TNF‐α), interleukin (IL)‐1α and IL‐10. Aspartate transaminase (AST), alanine transaminase (ALT) and urea levels were measured using spectrophotometry to ensure the safety of MSC therapy.

**Results:**

Gene expressions for all three inflammatory markers were reduced in both MSCs groups compared to the control group. AST, ALT and urea levels remained in normal ranges.

**Conclusions:**

MSC therapy can reduce inflammation in HCC xenograft mouse models.

## Graphical abstract

- Hepatocellular carcinoma (HCC) is the fifth most diagnosed cancer and the second leading cause of cancer‐related deaths in the world.
- Cell therapy with mesenchymal stem cells (MSCs) has proved effective in regulating the immune system and inflammatory responses in addition to preventing tumour growth.
- MSCs through their anti‐inflammatory effects can control HCC tumour growth.

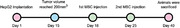

## 1 INTRODUCTION

Hepatocellular carcinoma (HCC) is a leading cause of cancer mortality worldwide (Hajighasemlou et al., 2020) which commonly develops following a prolonged chronic hepatitis infection (Berasain et al., 2009; Coussens & Werb, 2002). The progressive increase in HCC incidence and mortality is mainly due to viral hepatitis B and C. Despite recent advances in diagnostic tools, most HCCs are still first diagnosed in advanced stages where curative treatments are no longer an option (Lencioni et al., 2010).

Human multipotent mesenchymal stem cells (MSCs) have been isolated from various adult tissues, such as bone marrow, skeletal muscle, synovium, dental pulp, liver, brain, placenta, umbilical cord and adipose tissue (Campagnoli et al., 2001; Kim et al., 2010). Lacking immunogenicity, robust immunosuppressive and anti‐inflammatory properties, promoting angiogenesis and reducing apoptosis are fascinating features that have prompted their wide application in regenerative medicine (Boomsma & Geenen, 2012; Garcia‐Castro et al., 2008; Sanchez et al., 2011). In addition, MSCs can produce a high level of indoleamine 2,3‐dioxygenase (IDO1), a tryptophan catabolising enzyme that mediates immune tolerance by limiting the availability of the essential amino acid tryptophan and generating toxic metabolites for T cells (Meisel et al., 2004). Considering the properties above, we hypothesised that MSCs would effectively modulate the immune response in malignancies.

According to the Mesenchymal and Tissue Stem Cell Committee of the International Society for Cellular Therapy, the minimal criteria to standardise the isolation and expansion of MSCs include plastic adherence; expression of cluster of differentiation CD73, CD90 and CD105; lack of expression of CD14, CD34, CD45, CD70α or CD19 and human leukocyte antigen (HLA)‐DR surface molecules and the capacity to differentiate into osteoblasts, adipocytes and chondroblasts in vitro (Dominici et al., 2006).

This study aimed to investigate the anti‐inflammatory effect of MSCs on HCC xenografts in athymic nude mice.

## 2 MATERIALS AND METHODS

### 2.1 Cell culture

Human hepatocellular carcinoma cell lines (HepG2) were purchased from the Iranian National Center for Genetic and Biologic Resources (Tehran, Iran) and were cultured in Roswell Park Memorial Institute (RPMI)‐1640 media supplemented with foetal bovine serum (10%), penicillin (100 U/ml) and streptomycin (100 μg/ml) in standard conditions (37°C, 5% CO_2_ and 95% humidity). Human placenta‐derived MSCs were isolated from a single healthy donor (Oliveira & Barreto‐Filho, 2015), cultured in high‐glucose Dulbecco's Modified Eagle Medium (DMEM) under the standard condition. For MSCs identification, flow cytometry analysis was performed on the early passage MSCs assessing CD105, CD44, CD45, CD73, CD90 and CD34.

### 2.2 Xenograft models

A total of 12 male athymic nude mice (nu/nu; C57BL/6), aged 6–8 weeks, were purchased from Omid Institute for Advanced Biomodels (Tehran, Iran). The mice were housed and maintained in an individually ventilated cage system under optimised hygienic conditions. The average temperature of each cage was 23°C with a relative humidity of 65%. Animals had free access to autoclaved commercial diet and water. The study was approved by the Institutional Ethics Committee (ethical approval no. IR.TUMS.VCR.REC.1395.287).

**FIGURE 1:**
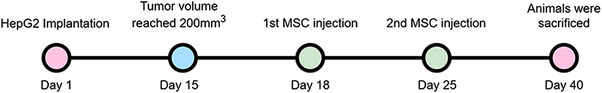
Timeline showing the sequence of events for the experimental groups. MSC, mesenchymal stem cell

HepG2 cells (1 × 10^7^) were suspended in 100 μl of serum‐free medium supplemented with 100 μl Matrigel (Corning, USA) and subcutaneously injected into the right and the left flanks of each mouse. Tumour dimensions were measured three times per week for each mouse using a vernier calliper; tumour sizes were calculated by the following formula: length × width^2^ × 0.52 (Figure 1). The animals were randomised into three groups to receive phosphate‐buffered saline (PBS) (the control group), intravenous MSCs (the IV group) or local MSCs (the local group). The treatment was initiated when the average volume of tumours among the 12 subjects had reached 200 mm^3^ (day 15). Both groups received human placenta‐derived MSCs (5 × 10^5^) either via tail veins (intravenous) or into the tumour margin (local) on days 18 and 25 post‐implantation. The mice were sacrificed 40 days post‐implantation (Figure 1), and the tumours were excised. 1 ml per 1000 mm^3^ of RNAlater (Qiagen, Germany) was added to store tissue specimens.

### 2.3 Analysis of biochemical factors

Blood samples were collected and centrifuged at 800 relative centrifugal force (RCF) to separate the plasma. The levels of aspartate aminotransferase (AST), alanine aminotransferase (ALT), and urea were determined using an automated biochemical analyser (Mindray) to assess the safety of the intervention.

### 2.4 Primer design for quantitative real‐time polymerase chain reaction (qRT‐PCR)

All gene sequences were obtained from the National Center for Biotechnology Information (NCBI) database, and primers were designed using Primer‐BLAST online software (NCBI).

### 2.5 qRT‐PCR analysis

qRT‐PCR was used to quantify the gene expression of inflammatory markers, including interleukin‐1a (IL‐1α), IL‐10 and tumour necrosis factor‐α (TNF‐α). Tumour tissue cells were harvested and total RNA was isolated using the RNeasy Plus Mini Kit (Qiagen, Germany), according to the manufacturer's instructions. Complementary DNA (cDNA) was synthesised using the First Strand cDNA Synthesis Kit (Takara Bio Inc., Japan). qRT‐PCR was conducted with the 7500 Real‐Time PCR System (Applied Biosystems®, Lincoln, CA) using Power SYBR® Green PCR Master Mix, 10 ng cDNA and specific primers (Table 1) in a total volume of 20 μl. Thermal conditions were the same for all genes and were cycled 46 times: initial denaturation at 95°C for 30 s, annealing at 60°C for 30 s and extension at 72°C for 35 s. Results were expressed as the cycle threshold (Ct) and were normalised to an internal control, glyceraldehyde‐3‐phosphate dehydrogenase (GAPDH), and calibrated against undifferentiated human endometrial stem cells (hEnSCs).

**TABLE 1:** List of primers used in the real‐time quantitative polymerase chain reaction (qRT‐PCR)

Gene name	Accession/version	Definition	Primer seq. 5'–3'
TNF	NM_000594.4	Homo sapiens tumour necrosis factor (TNF), mRNA	F: CCCGAGTGACAAGCCTGTAG
			R: TGAGGTACAGGCCCTCTGAT
IL1A	NM_000575.5	Homo sapiens interleukin 1 alpha (IL1A), transcript variant 1, mRNA	F: CTTCTGGGAAACTCACGGCA
			R: AGCACACCCAGTAGTCTTGC
IL10	NM_000572.3	Homo sapiens interleukin 10 (IL10), transcript variant 1, mRNA	F: GGCACCCAGTCTGAGAACAG
			R: ACTCTGCTGAAGGCATCTCG

Abbreviations: IL, interleukin; mRNA, messenger ribonucleic acid; TNF, tumour necrosis factor.

### 2.6 Statistical analysis

One‐way analysis of variance was applied to check the distribution of biomarkers, and after that, Dennett's test was performed in case of significant results. SPSS software was used to analyse the data, and the 2^–ΔΔCT^ method was used to calculate the relative transcript level. qRT‐PCR results were analysed by the Rotor‐Gene Q software using the Kruskal‐Wallis H test.

## 3 RESULTS

### 3.1 Analysis of biochemical factors

Serum AST, ALT and urea levels were in their normal ranges, with no statistically significant difference between groups (Table 2).

**TABLE 2:** Plasma levels of biochemical markers of the liver and kidney function to assess the safety of cell therapy

Group	Biochemical marker		
	AST	ALT	Urea
Control	367.75 ± 15	69.6 ± 3	58 ± 5.4
IV MSC injection	377 ± 37	68.5 ± 12	52.5 ± 3
Local MSC injection	338 ± 24	66.5 ± 7	56.3 ± 7.5
*p* Value	0.268	0.423	0.200

*Note*: No significant change was observed between the control and the treatment groups. All values are presented as mean ± SD.

Abbreviations: ALT, alanine transaminase; AST, aspartate transaminase; IV: intravenous; MSC, mesenchymal stem cell; SD, standard deviation.

### 3.2 qRT‐PCR

**FIGURE 2:**
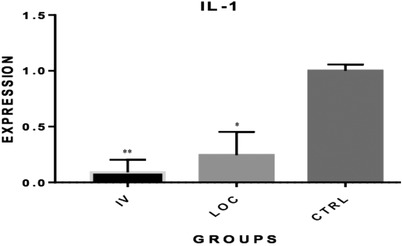
Comparison of TNF gene expression among groups. A statistically significant reduction was found in TNF expression in both systemic and locally administered groups as compared to control (***p < 0.001). MSC, mesenchymal stem cell; TNF, tumour necrosis factor; CTRL, control; IV, intravenously (systemically administered MSC); LOC, locally administered MSC (in tumour margin)

**FIGURE 3:**
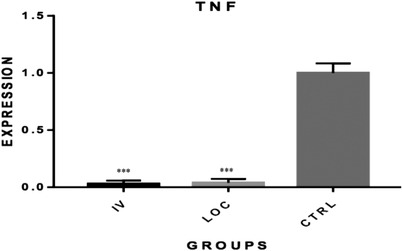
Comparison of IL‐1α gene expression among groups. A statistically significant reduction was found in IL‐1 expression in both systemic and locally administered groups when compared to control (*p < 0.05). IL‐1, interleukin‐1; MSC, mesenchymal stem cell; CTRL, control; IV, intravenously (systemically administered MSC); LOC, locally administered MSC (in tumour margin)

**FIGURE 4:**
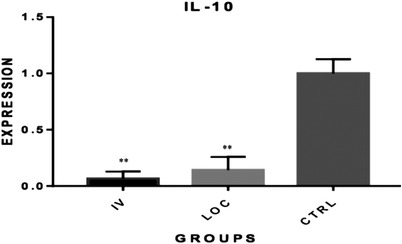
Comparison of IL‐10 gene expression among groups. A statistically significant reduction was found in IL‐10 expression in both systemic and locally administered groups when compared to control (**p < 0.01). IL‐10, interleukin‐10; MSC, mesenchymal stem cell; CTRL, control; IV, intravenously (systemically administered MSC); LOC, locally administered MSC (in tumour margin)

The qRT‐PCR results showed inhibition of IL‐1α, IL‐10 and TNF‐α expression in both intravenously and locally MSCs‐treated groups. The relative quantification of gene expression for IL‐1α was 0.09 for the IV and 0.24 for the locally administered MSC groups (*p* < 0.05; Figure 2). For IL‐10, these values equalled 0.07 and 0.014 for IV and locally administered MSC, respectively (*p* < 0.01; Figure 3). As for TNF‐α, the relative gene expression equalled 0.03 and 0.04 for IV and local groups, respectively (*p* < 0.001; Figure 4). The quantification for each marker was compared relative to controls.

### 3.3 MSC identification

CD105, CD44, CD45, CD73, CD90 and CD34 were assessed using flow cytometry. The results are presented in Figure S1.

### 3.4 Tumour size

**FIGURE 5:**
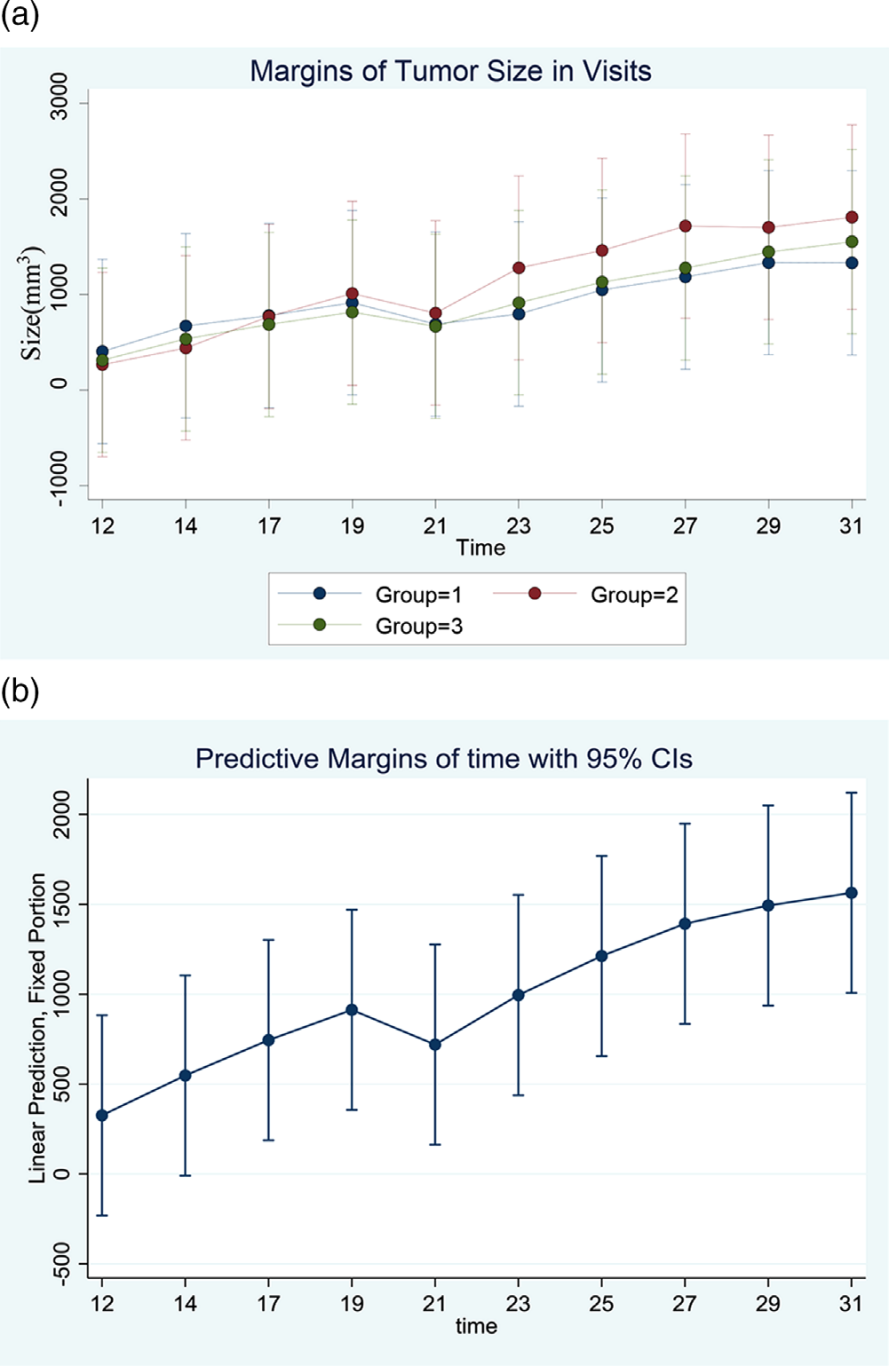
Change in tumour size is graphed across time. The tumour sizes were not significantly different among the three groups. (a) Margin of tumour size. (b) Predictive margin of time

The size of the tumours were also measured every 2–3 days after implantation. No significant difference was found among the average tumour sizes in different groups. Figure 5 depicts the changes in tumour size across time in all three groups.

## 4 DISCUSSION

One of the ways to investigate the anti‐inflammatory effect of MSCs on HCC xenografts is by determining the expression of various inflammatory mediators such as IL‐1α, IL‐2, IL‐4, IL‐8, IL‐10, TNF‐α and transforming growth factor‐β (TGF‐β) . In this study, we chose to investigate the expression of IL‐1α, IL‐10 and TNF‐α, due to their key role in immune regulation, inflammatory responses, tissue remodelling, cell motility, cell cycle and apoptosis (Malik & Kanneganti, 2018 and Kitaura et al., 2022).

In this study, we examined the effect of both systemic and local administration of MSCs on mouse models of HCC, where we found a reduction in three major inflammatory factors, namely, IL‐1α, IL‐10 and TNF‐α.

IL‐1α, as a pro‐inflammatory cytokine, induces a cascade of other pro‐inflammatory mediators, inflammatory cell infiltration and the expression of adhesion molecules on endothelial cells and leukocytes (Voronov et al., 2014). IL‐10, on the other hand, is an anti‐inflammatory molecule secreted mainly by activated B cells and macrophages. This effect is supported by the unregulated inflammatory response in IL‐10 knockout mice (Murray, 2006).

TNF‐α is an acute phase reactant involved in systemic inflammations with a significant role in tissue remodelling, cell motility, cell cycle and apoptosis. In liver fibrosis and alcoholic liver disease, TNF‐α acts by activating the c‐Jun N‐terminal kinase (JNK) pathway signalling cascade, thereby promoting cell survival through regulating c‐Jun, c‐Myc and p53 activities (Wang et al., 2013).

The abnormal expressions of both pro‐ and anti‐inflammatory mediators (e.g. IL‐1α, IL‐2, IL‐4, TNF‐α and TGF‐β) are responsible for many chronic liver disorders, including liver tumourigenesis. These factors exert their role by promoting c‐Myc gene expression, which can, in turn, upregulate the expression of IL‐8, IL‐10, TNF‐α and TGF‐β genes (Liu et al., 2015).

Expert opinions are controversial on the role of MSCs in inflammation and tumour progression (Voronov et al., 2014). However, it can be better considered by investigating the interplay between MSCs and the inflammatory milieu (Murray, 2006).

We have previously studied the homing of systematically and locally administered hPMSCs into HCC xenograft models and demonstrated the safety of hPMSCs. We also report the superior privilege of the locally administered cells to migrate to the tumour tissues (Hajighasemlou et al., 2018).

The commonly used medications for inflammation control include non‐steroidal anti‐inflammatory drugs (NSAIDs) and corticosteroids, which inhibit multiple inflammation enzymes and mediators. Anti‐inflammatory medications are also used in cancer treatment to control the symptoms and act as adjuvants for cytotoxic medications.

One mechanism by which MSCs can inhibit tumour growth is through the different mediators secreted by these cells, which is supported by a study on animal models of human hepatoma, where an increase in tumour cell apoptosis was observed through the down‐regulation of Bcl‐2, c‐Myc and proliferating cell nuclear antigen (PCNA) (Qiao et al., 2008).

In a study on mouse liver fibrosis models, a single intravenous administration of stem cells derived from human exfoliated deciduous teeth (SHEDs) removed the fibrotic scar. The researchers suggested that SHEDs serve this effect by suppressing the expression of pro‐inflammatory mediators (TNF‐α, IL‐1β and iNOS) and also by inducing apoptosis in hepatic stellate cells while selectively protecting parenchymal hepatocytes (Hirata et al., 2016).

Another cytokine secreted by MSCs is IL‐6 which acts as both pro‐inflammatory and anti‐inflammatory cytokines (Scherzad et al., 2015) studied the effect of MSCs on two head and neck cell lines (FaDu and HLaC78) via their interactions with cytokines; they found an inhibition in malignant cell proliferation when IL‐6 was added to the culture (Jiang et al., 2011). However, the inflammatory process in malignancies is relatively nonspecific, where we can see an increased expression of cytokines like IL‐4, IL‐5 and especially IL‐1α and TNF‐α, whereas the levels of IL‐2 are usually decreased (Baier et al., 2005). Taraxacum officinale (TO), commonly known as dandelion, has frequently been used to remedy women's diseases and livers’ and gallbladders’ disorders. Its anti‐tumour properties have also been elucidated in a study on the human hepatoma cell line, HepG2, where it induced apoptosis by increasing the levels of IL‐1α and TNF‐α in those cells (Koo et al., 2004).

Investigating the effect of microRNA‐22 on HCC, researchers detected an increased expression of IL‐1α in tumour‐adjacent tissues in the male subset, inversely correlated with the expression of oestrogen receptor α. Researchers further revealed that IL‐1α was higher in the early stages of tumourigenesis but decreased along with its development (Jiang et al., 2011).

## 5 CONCLUSION

Tumour progression can be adversely affected by unregulated inflammation; this property of tumour cells can benefit from controlling the milieu of inflammation using MSCs.

## CONFLICT OF INTEREST

Authors have no conflict of interest to declare.

## FUNDING

Tehran University of Medical Science and Cancer Research Center of Cancer Institute of Iran (Shams cancer charity) provided financial support.

## ETHICAL APPROVAL

Applied treatments in this study were approved by the Ethical Committee of TUMS.

## AUTHOR CONTRIBUTIONS

Saieh Hajighasemlou: Conceptualisation; formal analysis; writing – review & editing. Mohsen Nikbakht & Saeedreza Pakzad: Software; writing – review & editing. Abdolnaser Azadbakht & Milad Mirmoghtadaei: Methodology; validation; writing – review & editing. Samad Muhammadnejad & Iman Seyhoun: Software; writing – review & editing. Safoora Gharibzadeh: Formal Analysis. Javad Verdi: Conceptualisation; funding acquisition; writing – original draft; writing – review & editing.

### PEER REVIEW

The peer review history for this article is available at https://publons.com/publon/10.1002/vms3.886.

## Supporting information

FIGURE S1 Characterisation of mesenchymal stem cells using flow cytometry: CD105, CD44, CD90 and CD34 were present on over 90% of the cells (a, b, e, f); CD45 was negative on over 90% of the cells (c) and CD73 was positive on almost 100% of the cells (d). CD, cluster of differentiation; FITC, fluorescein isothiocyanate; FL, fluorescence; H, height; PE, phycoerythrin.

## Data Availability

The data that support the findings of this study are available from the corresponding author upon reasonable request.
