# Supplementary figures and images for: Anti‐inflammatory effect of mesenchymal stem cells on hepatocellular carcinoma in the xenograft mice model
Source: Vet Med Sci. 2022 Jul 15;8(5):2086–91. doi: 10.1002/vms3.886 (PMC9514506; doi:10.1002/vms3.886)

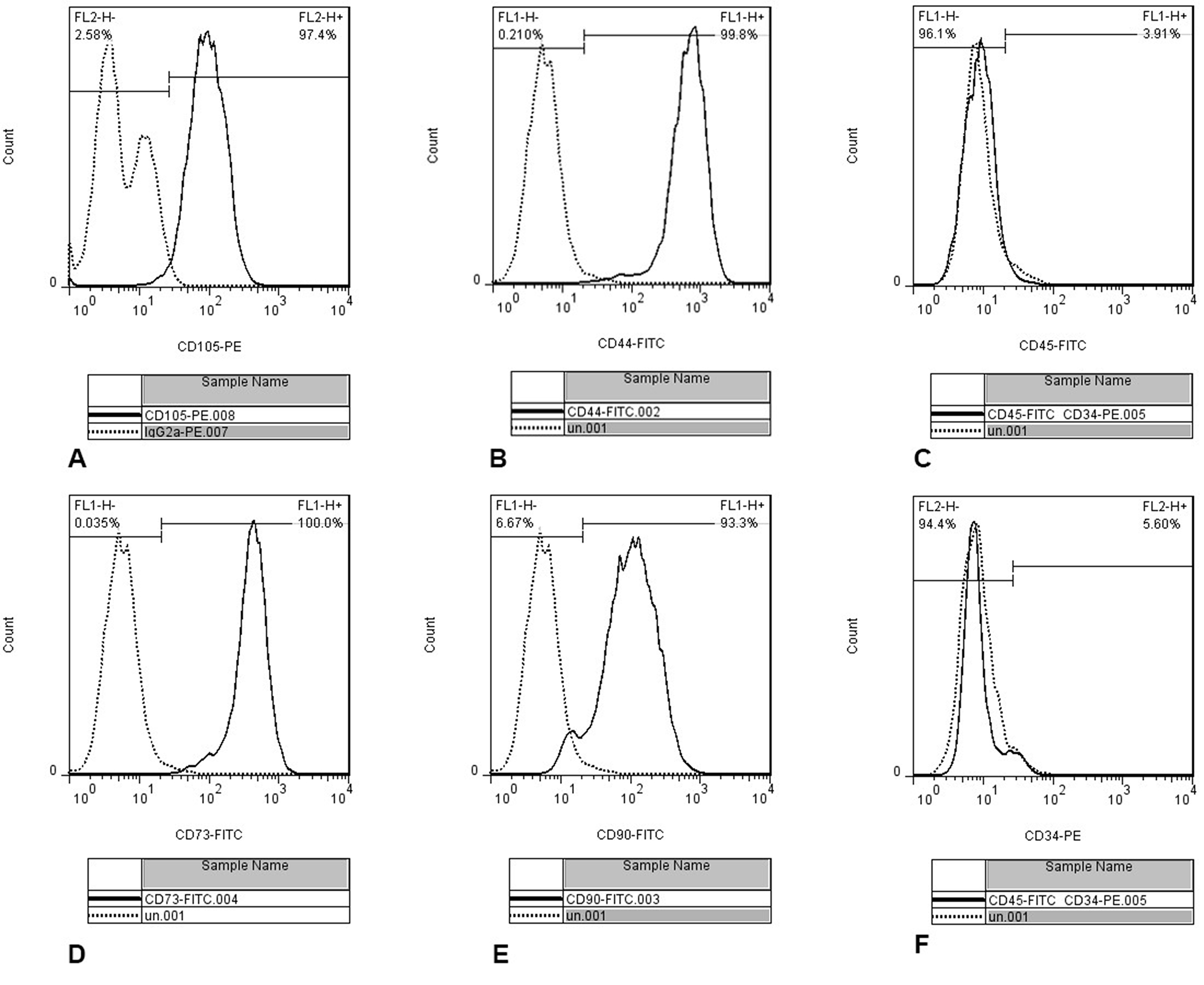

Supplement: Supplementary file 1 — FIGURE S1 Characterisation of mesenchymal stem cells using flow cytometry: CD105, CD44, CD90 and CD34 were present on over 90% of the cells (a, b, e, f); CD45 was negative on over 90% of the cells (c) and CD73 was positive on almost 100% of the cells (d). CD, cluster of differentiation; FITC, fluorescein isothiocyanate; FL, fluorescence; H, height; PE, phycoerythrin. [file VMS3-8-2086-s001.jpg]
